# Variation in Macronutrient Content, Phytochemical Constitution and *In Vitro* Antioxidant Capacity of Green and Red Butterhead Lettuce Dictated by Different Developmental Stages of Harvest Maturity

**DOI:** 10.3390/antiox9040300

**Published:** 2020-04-03

**Authors:** Christophe El-Nakhel, Antonio Pannico, Giulia Graziani, Marios C. Kyriacou, Maria Giordano, Alberto Ritieni, Stefania De Pascale, Youssef Rouphael

**Affiliations:** 1 Department of Agricultural Sciences, University of Naples Federico II, 80055 Portici, Italy; nakhel_christophe@hotmail.com (C.E.-N.); antonio.pannico@unina.it (A.P.); maria.giordano@unina.it (M.G.); depascal@unina.it (S.D.P.); 2 Department of Pharmacy, University of Naples Federico II, 80131 Naples, Italy; giulia.graziani@unina.it (G.G.); alberto.ritieni@unina.it (A.R.); 3 Department of Vegetable Crops, Agricultural Research Institute, 1516 Nicosia, Cyprus; m.kyriacou@ari.gov.cy

**Keywords:** harvest stage, *Lactuca sativa* L. var. *capitata*, chlorogenic acid, quinic acid, UHPLC-Q-Orbitrap HRMS, quercetin malonyl glucoside, lutein, β-carotene

## Abstract

Rising life expectancy and the demanding modern lifestyle drive the growing appeal of healthy and balanced diets centered on vegetable and fruit consumption. Functional, phytonutrient-packed and principally raw food is in high demand. Microgreens constitute such a novel functional food that combines a high sensory and bioactive value, which invites comparison to their mature-leaf counterparts. For this purpose, a controlled environment chamber experiment was carried out to compare the mineral, phytochemical and antioxidant capacity attributes of two-pigmented *Lactuca sativa* L. var. *capitata* cultivars (green and red Salanova^®^) harvested at the microgreens and the mature-leaf stage. Macronutrients were assessed through ion chromatography, while carotenoids and polyphenols were assessed and quantified through HPLC-DAD and UHPLC-Q-Orbitrap HRMS, respectively. Calcium and magnesium were higher in microgreens irrespective of the cultivar; conversely, phosphorous, potassium and nitrate where higher in mature leaves. All pigments including chlorophyll, lutein and β-carotene augmented at advanced maturity stage and were more concentrated in the red pigmented cultivar at both stages. Total polyphenols accumulated more densely in red Salanova, particularly in the microgreens stage; whereas, in green Salanova, the accumulation was significant but less pronounced in the mcirogreens stage. Chlorogenic acid, quercetin malonyl glucoside, rutin and coumaroyl quinic acid were the most concentrated phenolic acids in microgreens, while feruloyl tartaric acid was predominant in mature leaves. Finally, when a high carotenoids content is sought, mature lettuce leaves should be the prime culinary choice, whereas high polyphenolic content is dictated by both the cultivar and the harvest stage, with red Salanova microgreens being the most nutrient-packed choice.

## 1. Introduction

Nature commands a safe of an impressive repository of bioactive and potentially therapeutic ingredients [1,2,3]. In fact, a plethora of actual blockbuster medicines or their precursors have been isolated from natural sources [1,3]. In many countries of the developing world, ethnobotanical cures constitute the principle pharmaceutical resources exploited [1]. Indigenous populations rely largely on medicinal plants as remedies and edible plants for health support [2], while in the rest of the world plants are demonstrating a huge comeback in the context of healthier lifestyles, particularly as functional foods endowed with bioactive phytochemicals [4]. Driven by these emerging trends in human nutrition connected to physical well-being and nature’s legacy as provider of healthier and nutritional foods [2,4,5,6], agriculture is switching from cultivation for food to cultivation for health. Aside from primary metabolites, plants manufacture innumerable compounds via the secondary metabolism. These secondary metabolites, or better-called “ecochemicals”, are not essential for plant cells but they expose crucial features of the plant metabolome and partake in roles in plant survival, reproduction and interaction with the environment [3,7,8,9]. Derived from the primary metabolism [8,9], secondary metabolites command medical and socio-economic significance especially valued in scientific research and the polyphenols industry [3,8]. The 20th century witnessed diverse physicochemical methods allowing the characterization of a surfeit of complex molecular structures with antioxidant potential, whereas present advances in chromatography and identification tools such as mass-spectrometry enable us to separate, identify and characterize complex compounds with elevated molecular weight, low stability or trace concentrations [10].

Of these metabolites, phenolic compounds are primarily present in medicinal plants, herbs, vegetables and fruits [11], and are accountable for the bitterness, astringency, color and scent of these products [8]. Vegetables are reckoned as an indispensable integrant of human diet due to their ramified colors and beneficial health effects directly correlated with their molecular constituents’ antioxidant capacity. Leafy vegetables are widely consumed, mostly raw, all over the world [12]. Moreover, microgreens have presented an upcoming gastronomic component of culinary novelties in recent years [5,13,14,15], distinguished for their sensory value but also their dense phytochemical content [4,16]. However, few studies have provided comparative evidence of microgreens’ phytochemical content as opposed to their mature-leaf counterparts. In view of recent research based on human trials asserting that dietary antioxidants are crucial in regulating responses to inflammation and the immune system at cytological level [17], the increasing consumption of microgreens as phytochemically dense greens [18] invites a closer examination of their comparative composition compared to mature greens. Thus far, few works have addressed the differences in phytonutrient composition between mature plants and microgreens, the majority of which posited that the latter is nutritionally more valuable for human consumption, such as Pinto et al. [19], Huang et al. [13], Charleboi [17], Kyriacou et al. [6,14,15]. To our knowledge, only Pinto et al. [19] and Weber [20] compared the mature leaves of lettuce with lettuce microgreens and they addressed solely the comparative mineral profiles.

Based on the aforementioned factors, the aim of our work was to examine the differences between green and red-pigmented lettuce cultivars at two different harvest stages: microgreens and mature leaves. For this purpose, a growth chamber experiment under controlled climatic conditions was conducted in order to assess the influence of the developmental stage at harvest on the phenolic profiles and antioxidant attributes of green and red Salanova lettuce.

## 2. Materials and Methods 

### 2.1. Lettuce Cultivars and Climate-Chamber Conditions

Salanova Verde^®^ and Salanova Rossa^®^ (Rijk Zwaan, Der Lier, The Netherlands), two butterhead lettuce cultivars (*Lactuca sativa* L. var. *capitata*) with different leaf pigmentations, were chosen to assess their bioactive compounds and mineral content at two different harvest stages (Supplementary Figure S1). The adopted sowing and plantation density were 44,000 and 15 seeds m^−2^ for microgreens and mature lettuce, respectively. The experiment was performed in a 28 m^2^ walk through open-gas-exchange climate chamber (Process-C5, Spagnol srl, Treviso, Italy) (7.0 × 2.1 × 4.0 m; W × H × D), located at the experimental farm of the Department of Agriculture, University of Naples Federico II, Italy. Light was provided by High Pressure Sodium lamps (Master SON-T PIA Plus 400 W, Philips, Eindhoven, The Netherlands), having an intensity of 420 ± 10 μmol m^−2^ s^−1^ at canopy level and a light regime set at 12/12 h photoperiod. Climate chamber temperature was set at 24/18 °C (light/dark) with a corresponding relative humidity of 65%/80%, which was maintained by two HVAC (Heating, ventilation and air conditioning) systems. An organic substrate was used to fill the trays (56 × 37 × 5.5 cm: W × L × D) and the pots (8 × 8 × 7 cm) of microgreens and mature lettuce, respectively. Three replicate seeding trays were used for each microgreen cultivar, while for mature lettuce five plants per replicate per cultivar were grown.

Lettuce seeds were germinated in vermiculite, and then transplanted after 20 days in pots, where they were fertigated with a modified Hoagland formulation (9.0 mM N-NO_3_^−^, 2.0 mM S, 1.0 mM P, 4.0 mM K, 4.0 mM Ca, 1.0 mM Mg, 1.0 mM NH4^+^, 15 µM Fe, 9 µM Mn, 0.3 µM Cu, 1.6 µM Zn, 20 µM B, and 0.3 µM Mo), resulting an electrical conductivity (EC) of 1.5 dS m^−1^ and a pH of 6.0 ± 0.2. Lettuce microgreens were sown directly in the trays and fertigated with a quarter strength of the macro-elements used in the precedent nutrient solution and the same amount of the micro-elements, with an EC of 0.4 dS m^−1^.

### 2.2. Sampling and Fresh Yield of Microgreens and Lettuce Heads

The microgreens of green and red Salanova samples were harvested 16 days after sowing (DAS), at the appearance of the second true leaf, by cutting just above substrate level. Instead, the lettuce heads of green and red Salanova were harvested 19 days after transplanting (DAT). Fresh yield in both cases was expressed in kg m^−2^. Upon harvesting, fresh samples from each replicate at both harvest stages were collected arbitrarily and instantly frozen in liquid nitrogen then stored at −80 °C before being lyophilized in a Christ, Alpha 1–4 (Osterode, Germany) in order to be used for phytochemical analyses. 

### 2.3. Dry matter, Nitrate and Mineral Content 

Green and red butterhead Salanova samples at both harvest stages, were also oven dried at 70 °C for three days (until reaching constant weight) and weighed on an analytical balance (Denver Instruments, Denver, CO, USA) to get the dry weight. In order to determine dry matter (DM) percentage, the following formula was used: DM = 100 × Dry weight/Fresh weight. 0.25 g of the ground material was analyzed by ion chromatography (ICS-3000, Dionex, Sunnyvale, CA, USA) to determine the mineral content according to the method proposed by Rouphael et al. [21]. The results were expressed as mg 100 g^−1^ fresh weight (fw), whereas nitrate was expressed as mg kg^−1^ fresh weight (fw), according to each sample DM.

### 2.4. Chlorophylls and Total Ascorbic Acid 

Total chlorophyll content and chlorophylls a and b were determined according to Lichtenhaler and Wellburn [22]. Briefly, 500 mg of fresh leaves were ground in 10 mL of acetone (90%), then centrifuged at 3000× *g* for 10 min. The supernatant was collected and acetone (90%) was added again until reaching a volume of 25 mL. The Absorbance was measured using a spectrophotometer (Hach DR 2000, Hach Co. Loveland, CO, USA) at two-wave lengths 662 and 645 nm for chlorophylls a and b, respectively. Then total chlorophyll was calculated as the sum of chlorophylls a and b, and expressed in mg 100 g^−1^ fw. 

Total ascorbic acid (TAA) was assessed by spectrophotometric analysis of fresh Salanova material as described by Kampfenkel et al. [23] protocol. The quantification was assessed by UV–Vis spectrophotometry (Hach DR 4000; Hach Co., Loveland, CO, USA) at 525 nm, and the values were expressed as mg AA 100 g^−1^ fw.

### 2.5. Carotenoids Separation and Quantification by HPLC-DAD

As detailed in Kyriacou et al. [5], carotenoids (lutein and β-carotene) were extracted from lyophilized material in ethanol containing 0.1% butylated hydroxytoluene (BHT) as a modified method of Kim et al. [24]. A Shimadzu HPLC Model LC 10 (Shimadzu, Osaka, Japan) equipped with a reverse phase 250 × 4.6 mm and 5 µm Gemini C18 column (Phenomenex, Torrance, CA, USA) was used to separate carotenoid molecules. Absorbance was measured at 450 nm and quantification was executed against linear calibration curves done with lutein and β-carotene external standards, with concentrations ranging from 5 to 100 g mL^−1^. The results were expressed as mg kg^−1^ dw. A representative HPLC-Diode array chromatogram of carotenoids extracted from butterhead lettuce Salanova monitored at 450 nm is reported in Figure S2.

### 2.6. Polyphenols Extraction and Analysis by UHPLC-Q-Orbitrap HRMS

As described by Kyriacou et al. [6] 100 mg of lyophilized Salanova material was used to extract polyphenols. Briefly, these latters were quantified and separated through an UHPLC system (UHPLC, Thermo Fisher Scientific, Waltham, MA, USA) equipped with a Dionex Ultimate 3000 Quaternary pump and a thermostated Kinetex 1.7 µm biphenyl (10 × 2.1 mm) column (Phenomenex, Torrance, CA, USA). The Mass spectrometry analysis was facilitated by a Q Exactive Orbitrap LC-MS/MS (Thermo Fisher Scientific, Waltham, MA, USA). Polyphenolic compounds were acquired on parallel reaction monitoring mode [5]. The accuracy and calibration of the Q Exactive Orbitrap LC-MS/MS was monitored by using a Thermo Fisher Scientific reference standard mixture. Xcalibur software v. 3.0.63 (Xcalibur, Thermo Fisher Scientific, Waltham, MA, USA) was used to perform data analysis and processing. Retention time and m/z ions selected for the identification and confirmation of the target compounds in the Orbitrap system are presented in Table S1. The results were expressed as µg g^−1^ fw based on each sample DM; each result indicates the mean of six values (three replicates for each sample, injected two times). Representative Total Ion Chromatogram (TIC) of polyphenolic compounds extracted from butterhead lettuce Salanova evaluated by UHPLC-ESI-Orbitrap-MS is reported in Figure S3.

### 2.7. UHPLC-Q-Orbitrap HRMS Method Validation

The method for quantitative analysis of polyphenols was validated in order to evaluate linearity, intra-day precision, inter-day precision, limits of detection (LODs), limits of quantification (LOQs) and accuracy following the Directorate-General for Health and Food Safety (SANTE) guidelines (SANTE/11813/2017). The linearity of the method was evaluated at both low (LOQ—5 mg kg^−1^) and high (5–120 mg kg^−1^) concentration ranges, using eight concentration levels in each calibration range. Calibration curves for all compounds were prepared in triplicate. The linearity of the calibration curves was evaluated by the determination coefficient (*R*^2^). The method precision (intra and inter-day) was estimated by a triplicate determination at three concentrations (1, 10 and 50 mg kg^−1^) on the same day (intra-day) and on three non-consecutive days (inter-day), and expressed as percentage relative standard deviation (%RSD). LODs were defined as the minimum concentration for which the signal detected for the molecular ion (identified with mass accuracy <5 ppm) was higher than 1 × 10^3^ counts. LOQs were established as the minimum concentration for which the error mass was lower than 5 ppm and precision <20%. Accuracy is based on recovery studies, which were performed by spiking, in triplicate, pre-analyzed samples at 1, 10 and 50 mg kg^−1^. As a blank, the lettuce matrix could not be obtained, therefore the standard addition technique was employed. 

### 2.8. Statistical Analysis and Principal Component Analysis

The interaction between the two factors (Cultivar-C and Harvest stage-H) was subjected to ANalysis Of VAriance (Two-way ANOVA), while the mean effect of C and H were compared by *t*-Test using the software package SPSS 20. C × H interaction was assessed by Duncan’s Multiple Range Test (DMRT) performed at *p* ≤ 0.05. Principal component analysis (PCA) was carried out on macronutrient content, pigments, total ascorbic acid, target carotenoids and polyphenols, to depict the nutritional and functional quality aspects that were most effectual in distinguishing among butterhead lettuce cultivars (green and red-pigmented) and harvest stage (microgreens and mature leaves) by employing Minitab 16.2.1 statistical software. Calculations and analyses were also carried out using the appropriate functions within SPSS 20 software package to determine the score plot and loading matrix based on the first and second principal components (PC1 and PC2).

## 3. Results and Discussion

### 3.1. Yield and Dry Matter of Microgreens and Mature Salanova

**Figure 1:**
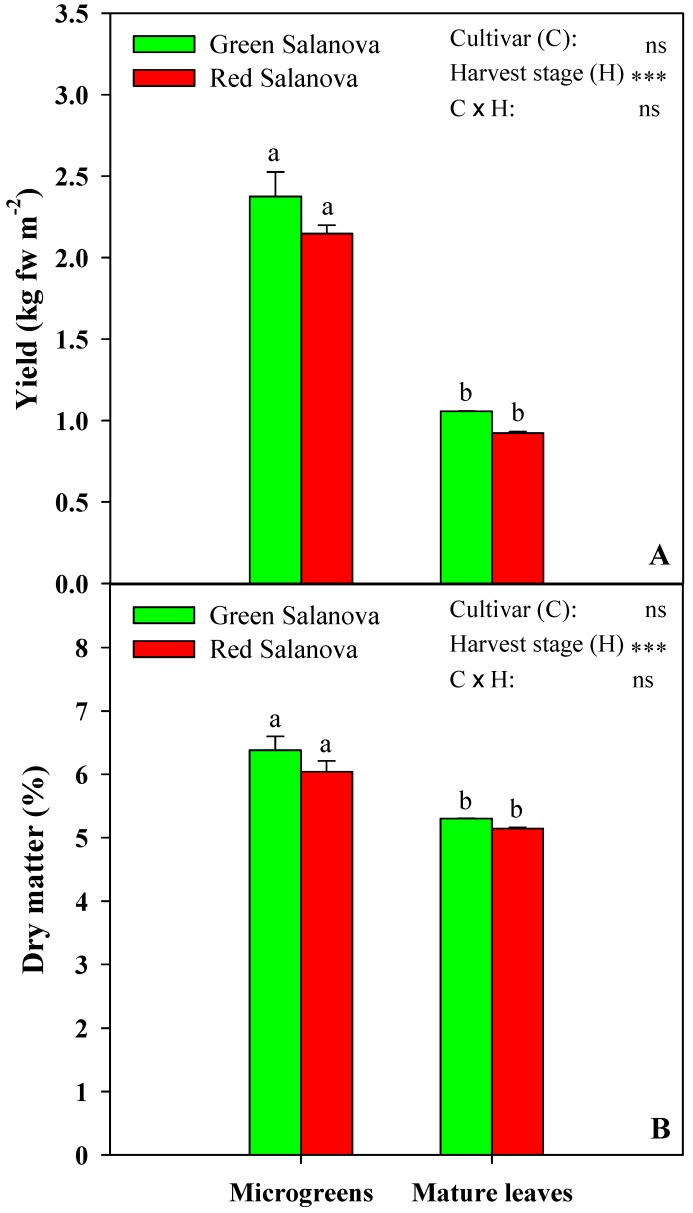
Yield (**A**) and dry matter (**B**) of green and red Salanova butterhead lettuce as dictated by different developmental stages of harvest maturity. ns, *** Non-significant or significant at *p* ≤ 0.001, respectively. Cultivar and harvest stage means were compared by *t*-test. Different letters indicate significant differences according to *t*-test (*p* = 0.05). All data are expressed as mean ± standard error, *n* = 3.

As portrayed in Figure 1A,B, respectively, yield and dry matter (DM) percentage of green and red Salanova showed no significant interaction between cultivar and harvest stage. Microgreens registered on average a yield of 2.27 kg fw m^−2^, which was significantly higher (2.3-fold) than that of mature lettuce, with green Salanova having a slightly and non-significantly higher yield at the microgreens and mature stages. The double yield of microgreens compared to mature leaves was expected due to the difference in sowing density, with 44,000 plants m^−2^ for microgreens versus 15 plants m^−2^ for mature leaves. Dry matter also exhibited significant variation with regards to harvest stage only, as mature leaves registered 5.2% DM, which was around 16.0% less than that of microgreens. In a similar work by Pinto et al. [19], where lettuce was compared at microgreens and mature stages, moisture content had remained almost equal. This could be attributed to the fact that this experiment was carrried out in a greenhouse with natural sunlight in winter, where microgreens had received less light intensity, especially at sunrise and sunset, which led the plants to expand their leaf area more in order to capture more photons, and thus created a dilution effect, compared to our microgreens, which were grown in a controlled growth chamber, with a steady photon flux during the entire 12 h photoperiod, that led to a compact microgreens shape with greater content of DM.

### 3.2. Nitrate Concentration and Mineral Content of Microgreens and Mature Salanova

Nitrate concentration, P, K, Mg and Na contents showed neither a significant interaction between the cultivar and the harvest stage nor a significant difference among the cultivars (Table 1). With respect to harvest stage, nitrate registered a concentration of 93 mg kg^−1^ fw in microgreens, 16-fold less than mature leaves, while P registered a content of 12.29 mg 100 g^−1^ fw, 35.7 % less than its mature counterpart. As for Mg content, it was 2-fold higher in mature leaves. Moreover, Na registered a content of 32.99 and 4.44 mg 100 g^−1^ fw in microgreens and mature leaves, respectively. As for K, mature leaves accumulated 3.5-fold more than microgreens lettuce (Table 1). On the other hand, Ca and S concentrations exhibited significant interaction between the cultivar and the harvest stage as shown in Table 1. Red Salanova microgreens was significantly the richest in terms of Ca between all treatments; red mature leaves had the least content of Ca, whereas green mature leaves had the least content of S. Overall, lettuce microgreens were richer in Ca, Mg, S and Na, whereas, mature leaves where richer in nitrate, P and K. 

Minerals take a crucial part in versatile biological processes during plant growth stages and development, where up to 17 key minerals take part, which are transferred to human nutrition [25]. These major minerals in human diet are of utmost importance to avert any disorders, their absence affect body homeostasis and metabolism [5]. Kyriacou et al. [5] stated that potassium is the most concentrated macronutrient in microgreens followed by calcium and magnesium. In fact, potassium is abundant in plant cells and tissues, calcium partakes in coordinating plant response to internal and external stimuli and magnesium is involved in chlorophyll and protein synthesis [25]. Refering to lettuce, Weber [20] showed that hydroponically grown romaine lettuce had higher P, Mg and S and lower K, Ca and Na than mature lettuce, which is not completely in line with our results, noting that in this previous experiment mature romaine lettuce were bought from the store and the cultivar was not defined. However, Pinto et al. [19] had comparable results to ours regarding macronutrients and nitrate, which were 4-fold higher in mature lettuces. However, nitrate in both Salanova’s cultivar and harvest stages, as presently examined, did not exceed the European Commission maxima [26] set at 3000–5000 mg kg^−1^ fw for lettuce, with lettuce microgreens surprisingly accumulating very low amounts (± 93 mg kg^−1^ fw). In fact, when commercial maturity is closer, nitrate concentration rises, due to leaves’ self-shading which reduces light incidence and nitrate reduction [27], which reflects the higher value in our mature leaves compared to microgreens lettuce. On the other hand, the sodium concentration in the lettuce microgreens studied in our work is expected, since in the previous works of Kyriacou and coworkers [5,6] on 17 different species and cultivars of microgreens, sodium ranged from 0.66 in amaranth to 12.02 g kg^−1^ dw in Mizuna. Similarly, low values of sodium in mature leaves are in line with the results obtained by El-Nakhel et al. [9] when green and red Salanova where cultivated in hydroponics under the same climate conditions. Anyhow, in this latest work sodium leaf content increased when potassium leaf content decreased, which is somehow the case in our experiment, where potassium and sodium are negatively correlated. This could be explained by the subsitution of K shortage by Na in the maintenance of vacuolar osmotic potential [9]. As for the increase of K content in mature leaves, this is explained by the main role played by this mineral in potential-driven water uptake and turgor-drivern cell expansion [28]. Similarly, P is also richer in mature plants, due its crucial role in energy distribution and nucleic acid synthesis [29], whereas Ca’s higher content in mcirogreens might be due to its role in regulating many stress-responsive genes if we affirm that the microgreens stage is a stress condition stage that is also explained by a higher polyphenols content (Table 4), such as caffeic acid in case of red Salanova. This polyphenol is influenced by Mg, which explains the higher content of the latter in red microgreens Salanova compared to mature leaves [9]. Overall, microgreens seem to be a strategic substitute to reduce nitrate intake and increase essential minerals intake [19].

### 3.3. Pigments and Total Ascorbic Acid Content of Microgreens and Mature Salanova

Total chlorophyll, chlorophyll a and chlorophyll b exhibited a significant interaction between the cultivar and harvest stage (Table 2). At the microgreens stage, chlorophyll a was statistically the same among red and green Salanova, while chlorophyll b and total chlorophyll were 1.6 and 1.2-fold higher in red Salanova, respectively. In mature stage, chlorophyll a, chlorophyll b and total chlorophyll increased even more and were higher in red Salanova by 2-, 2.6- and 2-fold, respectively (Table 2). 

The non-destructive measurements of chlorophyll content (SPAD Index) [30] measured by El-nakhel et al. [31], reflected the changes obtained regarding chlorophyll pigment measurements in our work. Indeed, chlorophyll increased significantly with lettuce maturation, and in both harvest stages was significantly higher in red Salanova, which was the exact same trend observed with chlorophyll measurements of lettuce at 4 and 19 DAT [31]. In photosynthetic tissues, carotenoids, and chlorophylls a and b operate in light harvesting and perform tasks in photo-protection by quenching free radicals, singlet oxygen, and other reactive species [30]. In our case, this is expected with lettuce growth, accompanied by an increase in the plants leaf area, implicating a higher light interception by the plants and consequently a higher need of light harvesting and photo-protection. Moreover, chlorophyll has many health benefits, such as magnesium delivery and its utility in chelating calcium and heavy minerals, and aside vitamin A, C and E, chlorophyll collaborate in neutralizing free radicals from damaging healthy cells [32]. 

As for total ascorbic acid (TAA), part of our results confirmed what was found in a previous experiment [33], where green and red Salanova plants were cultivated in hydroponics and compared at six maturity stages from 4 till 19 DAT in a growth chamber. Similarly, microgreens green Salanova had an equal content of TAA (43.58 mg AA 100 g^−1^ fw) that decreased around 4-fold at the end of the cycle, whereas red Salanova mature leaves had an increase of 23.7% in comparison to red Salanova microgreens (44.36 mg AA 100 g^−1^ fw), while in hydroponics TAA significantly decreased during the cycle yet maintained a relatively high TAA compared to its green counterpart. Accordingly to microgreens red Salanova, ascorbic acid increased from amaranth microgreens stage to fully grown leafy amaranth [34]. However, phenols along with ascorbate serve as scavengers of reactive oxygen species (ROS) for protecting young expanding leaves that are prone to light damage [35], which can explain why TAA in green Salanova decreased from microgreens to mature stage. Ascorbic acid is a potent dietary water-soluble antioxidant, and like most vitamins, is not synthesized by humans, unlike animals and plants, therefore its input from exogenous sources is a requirement. A diet containing fruits and vegetables helps in procuring this vitamin [36,37], and leafy vegetables account for a significant input [31]. It notably diminishes the hostile effect of reactive nitrogen and oxygen species, scavenges free radicals in our body and has anti-carcinogenic and immunomodulatory effects. Furthermore, ascorbic acid can revitalize other antioxidant compounds like α-tocopheroxyl and β-carotene radical cations from their radical species [36], and can rejuvenate vitamin E [37]. 

### 3.4. Carotenoid Content of Microgreens and Mature Salanova

Carotenoids account for a versatile group of lipophilic pigments, where β-carotene is a hydrocarbon carotene and lutein is an oxygenated xanthophyll; the latter are the most profuse in lettuce [33,38]. Dietary intake of carotenoids reduces degenerative diseases like chronic eye impairments. In particular, β-carotene is a precursor of vitamin A that is a retina constituent and lutein protects the eyes by absorbing excessive light [15], and specifically filtering the blue light additionally to its anti-oxidant properties [39,40]. Noting that among the carotenoids, solely lutein was consistently related to a broad range of cognitive measures, of which we mention executive function, language, learning and memory [40].

A significant interaction was present between the cultivar and the harvest stage for lutein and β-carotene. They all increased along plant growth. As shown in Table 2, both lutein and β-carotene, increased more acutely in red mature leaves than in green ones when compared to microgreens stage. Lutein in red mature leaves registered a value of 1338 μg 100 g^−1^ fw, 2.4-fold higher than green mature leaves and 153.4 % higher than red microgreens. Similarly, β-carotene in red mature leaves registered a value of 2656 μg 100 g^−1^ fw, 1.8-fold higher than green mature leaves and 69.5 % higher than green microgreens. Carotenoids are naturally higher in red-pigmented leaves [41]. The increases in lutein and β–carotene are in line with the results of Moura et al. [42], which had a higher total carotenoids concentration in lettuce at 40 DAT compared to 20 and 30 DAT, and quoted from other authors that lettuce carotenoids concentration can increase three to four times with maturation. Likewise, β–carotene increased in fully grown leafy amaranth in comparison to microgreens amaranth [34]. 

**Figure 2:**
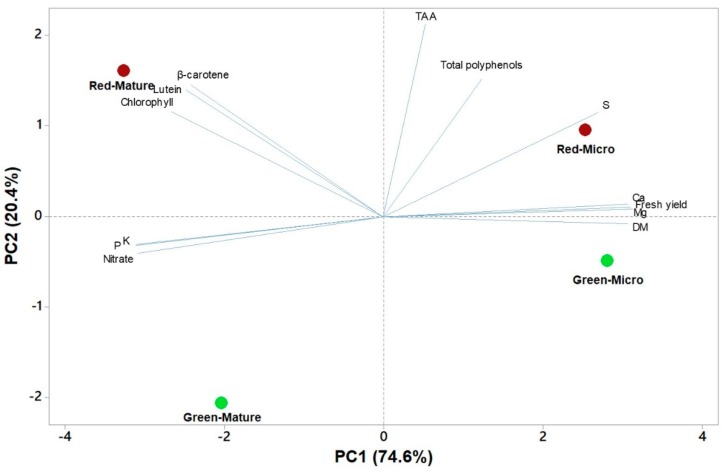
Principal component loading plot and scores of principal component analysis (PCA) of fresh yield, dry matter percentage (DM), macromineral (K, Ca, Mg, P, S and nitrate), chlorophyll, carotenoids (lutein and β-carotene) and total polyphenols in green and red microgreens and mature leaves of Salanova lettuce.

β–carotene is a complement to chlorophyll’s light-harvesting compound [43], and the synthesis of carotenoids is regulated by light [43], thus our mature lettuce with a higher leaf area (data not shown) which intercepted more light for a longer period of time in comparison to microgreens, most probably accumulated more lutein and β–carotene. Moreover, β-carotene and lutein contents were correlated (Figure 2), and both of these pigments also correlated with chlorophylls a, b and total chlorophyll, which correspond to the same trend seen in Mou [44] work on lettuce. The same author declared that carotenoids dietary intake is generally considered better than supplement intake. Moreover, Kim et al. [41] mentioned that pre-harvest factors such as “genetic makeup” and harvest stage of plants dedicate the nutrient composition of vegetables. As matter of fact, in his work regarding 23 baby leaf lettuce cultivars, red-leaf cultivars showed a distinguishing phytoconstituent profile, and cultivars were substantially rich in carotenoids, total phenolic content and anitoxidant potential, wich is the same case of our mature and microgreens red Salanova. 

### 3.5. UHPLC-HRMS Orbitrap Validation 

The obtained mass accuracy (the deviation of the measured accurate mass from the calculated exact mass of an ion), was below 5 ppm for all analytes (Supplementary Table S1). LODs were in the range from 0.03 to 0.05 μg kg^−1^ and LOQs from 0.1–0.16 μg kg^−1^ (Table 3). Correlation coefficients (r^2^) obtained for all investigated compounds were >0.990. Average recoveries from 91.6% to 105.9% for the 16 studied analytes were obtained at three spiking levels (1, 10 and 50 mg kg^−1^) (Table 3). The method precision (intra- and inter-day) showed an RSD of ≤10%, highlighting the overall precision of the proposed methodology (Table 3).

### 3.6. Polyphenols Profile of Microgreens and Mature Salanova

Phenolic coumpounds are omnipresent in plants and they present health-fostering benefits [10], which capture researchers’ attention. Moreover, lettuce represents a good source of phenolic compounds, with red cultivars being particularly rich [9,41]. In the current study, total polyphenol content showed a significant interaction between the cultivar and the harvest stage. As presented in Table 4, total polyphenols were significantly higher in the microgreens than the mature leaves stage for both green and red Salanova; they registered 36% and 106% higher content, respectively. In detail, as mentioned in Table 4, all polyphenols showed an interaction between the cultivar and the harvest stage, except for kaempferol-3-*O* rutinoside, which was significantly higher in microgreens. Feruloyl tartaric acid was not detected in the microgreens stage, while it registered a higher content in green mature leaves than in red ones and the most abundant phenolic acid in the green cultivar. However, dicaffeoylquinic acid, present in both green and red microgreens, was not detected in green mature leaves and decreased by 59% in red mature leaves in comparison to red microgreens. The most abundant polyphenols in red microgreens were: chlorogenic acid, quercetin malonyl glucoside, rutin, coumaroyl quinic acid and caffeoyl feruloyl quinic acid. The same polyphenols were also the most abundant in red mature leaves, but decreased by around 60%, except for caffeoyl feruloyl quinic acid which only decreased by 27%; notably, feruloyl tartaric acid that was not detected in the microgreens stage and was the fourth most abundant in red mature leaves. As for green microgreens, the most abundant polyphenols were: quercetin malonyl glucoside, rutin, caffeoyl feruloyl quinic acid, coumaroyl quinic acid and apigenin malonyl glucoside, while, for green mature leaves, the most abundant polyphenols were: feruloyl tartaric acid, rutin, quercetin malonyl glucoside, caffeoyl feruloyl quinic acid and coumaroyl quinic acid. 

This detected variability was stated by Imeh and Khokhar [11], who asserted that phenolic content fluctuates between different cultivars as well as tissues of the same fruit or vegetable. Moreover, in Kim et al. [41] red-leaf cultivars were rich in total phenolic content, as was the case at both harvest stages. These phenolic compounds have antioxidant potential, such as a major radical-quenching activity that confers a valuable nutritional asset to such vegetables in minimizing oxidative stress-related diseases. Our results are in line with Huang et al. [13], who obtained in his research more polyphenols in microgreens red cabbage than in mature red cabbage, such as 3-caffeoylquinic acid, caffeoyl glucose, sinapoyl–hexose and disinapoylgentiobiose. Equally, coriander microgreens exhibited a higher (47%) total phenols content in comparison to their mature counterparts [45]. In another work, rutin in coriander was a dominant phenolic acid in the microgreens stage [5], which is in harmony with our work. Chlorogenic acid was also highest among phenolic acids in Kyriacou et al. [6] and 10-fold higher in red amaranth and dark green purslane than in green mizuna and cress, the same as red Salanova in this work. Chlorogenic acid was higher in microgreens than in leafy green buckwheat, whereas rutin was not significantly different between the two maturity stages, but quercetin and total polyphenol content were higher in leafy greens’ buckwheat [46]. The latter finding forces us to avoid generalizing that microgreens have always better phyto-nutritional profile than their mature counterparts, especially when experiments are carried out in a natural environment with fluctuating external factors. Accordingly, Boudet [8] announced that several polyphenols take part in plant defense; these compounds shield against pests, while other simple phenolics are involved in adaptive tactics as deterrents. Such an interpretation could be also the answer to why young plants (i.e., microgreens) are usually rich in polyphenols; most probably, this is a basic survival strategy required to reach mature stages. Furthermore, the same author explained that quinic acid in angiosperms exhibited an accumulation pattern during intense growth. This accumulation resulted from an active photosynthetic activity, and such compounds serve as a carbon source for the synthesis of other phenolic compounds. Although this explanation was given for wooden plants, it could probably also explain why caffeoyl feruloyl quinic acid, coumaroyl quinic acid, dicaffeoylquinic acid, and feruloyquinic acid decreased in concentration from microgreens to mature stage in lettuce simultaneously with the reduction in the growth rate of our mature lettuce plants.

### 3.7. Principal Component Analysis of Functional and Nutritional Aspects of Green and Red Salanova at Microgreens and Mature Stages 

**Figure 3:**
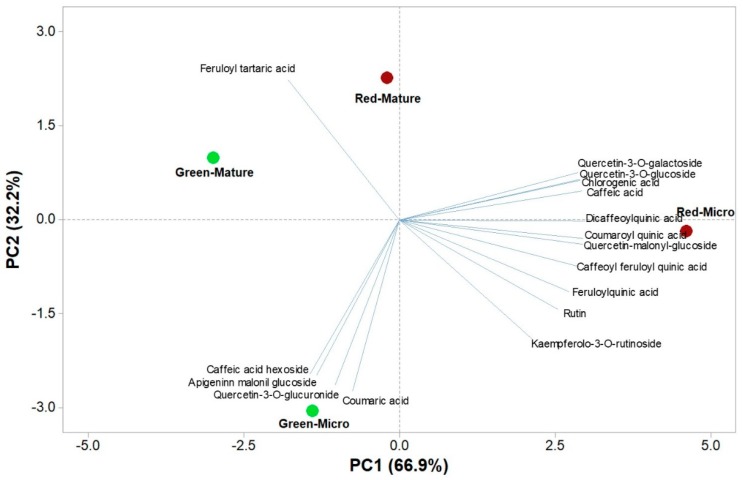
Principal component loading plot and scores of PCA of phenolic compounds quantified by HUPLC-Q-Orbitrap HRMS analysis in green and red microgreens and mature leaves of Salanova lettuce.

The principal component analysis (PCA) of yield, mineral profile, nutritional and functional traits as well as on the polyphenolic composition, are reported in Figure 2 and Figure 3. In the first PCA (on yield, mineral profile, nutritional and functional traits), the first two principal components (PCs) were related with eigen values higher than 1 and explained 95.1% of the total variance, with PC1 and PC2 accounting for 74.6% and 20.4%, respectively (Figure 2). Moreover, in the second PCA (on polyphenolic composition) the first two PCs were related with eigen values higher than 1 and explained 99.1% of the total variance, with PC1 and PC2 accounting for 66.9% and 32.2%, respectively (Figure 3). In the current study, the loading matrix indicates that variation in lutein and β-carotene was most closely aligned with total chlorophyll, whereas variation in nitrate was not correlated with total polyphenol content (Figure 2). The effectiveness of PCA in interpreting species/cultivars differences across several functional quality attributes in response to a wide range of preharvest factors such as the genetic material, the maturity stage, environmental conditions and agricultural practices have been reported previously by several researchers [5,6,9,31]. This was also the case of this study conducted under fully controlled conditions, since the score plot of PCA integrated information about the nutritional and functional profile of the red and green-pigmented lettuce harvested at microgreens or at mature stage (Figure 2 and Figure 3). Particularly, the red-pigmented microgreens were positioned on the positive side of PC1 in the upper right quadrant of the PCA score plot as it delivered leaves of premium quality with a high concentration of S, Ca, Mg, TAA and total polyphenols (Figure 2). Moreover, red-pigmented mature Salanova lettuce was characterized by higher pigments such as chlorophyll, β-carotene and lutein (Figure 2). The score plot of the second PCA integrated useful information on the polyphenolic profile of the tested red and green genotypes of microgreens and mature lettuce (Figure 3). The red-pigmented microgreens lettuce was positioned on the positive side of PC1 in the lower right quadrant of the PCA score plot as it delivered microgreens of premium quality with high concentration of dicaffeoylquinic acid, coumaroyl quinic acid, quercetin-malonyl-glucoside, caffeic acid and caffeoyl feruloyl quinic acid (Figure 3). Moreover, green microgreens lettuce were positioned in the lower left quadrant, characterized overall by a higher apigenin malonyl glucoside, coumaric acid, caffeic acid hexoside and quercetin-3-*O*-glucuronide (Figure 3).

## 4. Conclusions

The consumption of butterhead lettuce Salanova mature leaves vs. microgreens presents a win–win situation as long as the objective is a vegetable-rich diet that contributes bioactive compounds to the human diet. Irrespective of cultivar, Ca and Mg were more concentrated at the microgreens stage, while P and K were more concentrated at the mature leaves stage. Moreover, we demonstrated through profiling and quantification of carotenoids and polyphenols that antioxidant attributes exhibited diverse patterns between the two cultivars. Lutein and β-carotene content in mature leaves increased by 153% and 70%, respectively, in red Salanova, and by 86% and 43%, respectively, in green Salanova. While the opposite trend was demonstrated for polyphenols, where red and green microgreens content was 106% and 37% higher than that of mature leaves, respectively. In both stages, red pigmented lettuce performed better regarding lutein, β-carotene, total ascorbic acid and polyphenol content. Considering all treatments, red Salanova microgreens are a phytonutrient-packed produce convenient for urban cultivation, securing easy access to fresh greens and being low in undesirable elements such as nitrate, which are therefore a health-promoting culinary ingredient and not solely an edible garnish.

## Figures and Tables

**Table 1:** Nitrate concentration and macronutrient content of green and red Salanova butterhead lettuce as influenced by harvest stage.

Harvest Stage	Cultivar	Nitrate	P	K	Ca	Mg	S	Na
		(mg kg^−1^ fw)	(mg 100 g^−1^ fw)	(mg 100 g^−1^ fw)	(mg 100 g^−1^ fw)	(mg 100 g^−1^ fw)	(mg 100 g^−1^ fw)	(mg 100 g^−1^ fw)
Microgreens	Green	69 ± 3.3	12.33 ± 0.58	117.3 ± 2.33	52.48 ± 0.85 ^b^	23.62 ± 0.61	6.51 ± 0.20 ^a^	33.42 ± 1.39
	Red	117 ± 3.9	12.25 ± 0.44	127.4 ± 5.03	59.71 ± 0.88 ^a^	22.95 ± 0.60	6.85 ± 0.20 ^a^	32.56 ± 0.91
Mature leaves	*Mean*	*93 ± 11.0 ^B^*	*12.29 ± 0.33 ^B^*	*122.4 ± 3.36 ^B^*	*56.10 ± 1.71 ^A^*	*23.28 ± 0.41 ^A^*	*6.68 ± 0.15 ^A^*	*32.99 ± 0.77 ^A^*
	Green	1557 ± 40.8	18.94 ± 0.93	419.8 ± 8.18	29.17 ± 0.34 ^c^	12.49 ± 0.37	3.03 ± 0.03 ^c^	4.86 ± 0.18
	Red	1520 ± 31.3	19.28 ± 0.89	430.4 ± 3.76	21.85 ± 0.73 ^d^	10.50 ± 0.21	4.56 ± 0.26 ^b^	4.02 ± 0.12
	*Mean*	*1538 ± 24.4 ^A^*	*19.11 ± 0.58 ^A^*	*425.1 ± 4.67 ^A^*	*25.51 ± 1.68 ^B^*	*11.49 ± 0.48 ^B^*	*3.80 ± 0.36 ^B^*	*4.44 ± 0.21 ^B^*
Significance								
Cultivar (C)		ns	ns	ns	ns	ns	ns	ns
Harvest stage (H)		***	***	***	***	***	***	***
C × H		ns	ns	ns	***	ns	*	ns

All data are expressed as mean ± standard error, *n* = 3. ns,*, *** Non-significant or significant at *p* ≤ 0.05 and 0.001, respectively. Cultivar and harvest stage means were comparated by *t*-Test. Interaction between the two factors were compared by Duncan’s multiple-range test (*p* = 0.05). Lower case letters indicate the significant differences of the interaction, upper case letters indicate the significant differences of the harvest stage mean effect.

**Table 2:** Total ascorbic acid (TAA), chlorophyll, lutein and β -carotene content of green and red Salanova butterhead lettuce as influenced by harvest stage.

Harvest Stage	Cultivar	TAA	Chlorophyll ^a^	Chlorophyll ^b^	Total Chlorophyll	Lutein	β-carotene
		(mg AA 100 g^−1^ fw)	(mg 100 g^−1^ fw)	(mg 100 g^−1^ fw)	(mg 100 g^−1^ fw)	(μg 100 g^−1^ fw)	(μg 100 g^−1^ fw)
Microgreens	Green	43.58 ± 2.70 ^b^	5.15 ± 0.32 ^c^	1.01 ± 0.03 ^d^	6.16 ± 0.28 ^d^	300 ± 20 ^c^	1058 ± 97 ^c^
	Red	44.36 ± 2.69 ^b^	6.02 ± 0.57 ^c^	1.58 ± 0.10 ^c^	7.60 ± 0.60 ^c^	528 ± 50 ^b^	1567 ± 136 ^b^
Mature leaves	*Mean*	*43.97 ± 1.71*	*5.58 ± 0.35 ^B^*	*1.30 ± 0.14 ^B^*	*6.88 ± 0.44 ^B^*	*414 ± 56 ^B^*	*1313 ± 136 ^B^*
	Green	11.46 ± 0.18 ^c^	8.78 ± 0.12 ^b^	2.06 ± 0.11 ^b^	10.57 ± 0.09 ^b^	558 ± 48 ^b^	1516 ± 61 ^b^
	Red	55.12 ± 0.82 ^a^	17.80 ± 0.09 ^a^	4.64 ± 0.21 ^a^	22.48 ± 0.27 ^a^	1338 ± 36 ^a^	2656 ± 58 ^a^
	*Mean*	*33.29 ± 9.77*	*13.29 ± 2.02 ^A^*	*3.35 ± 0.59 ^A^*	*16.52 ± 2.66 ^A^*	*948 ± 176 ^A^*	*2086 ± 258 ^A^*
Significance							
Cultivar (C)		*	ns	ns	ns	*	*
Harvest stage (H)		ns	**	**	**	*	*
C × H		***	***	***	***	***	*

All data are expressed as mean ± standard error, *n* = 3. ns,*,**, *** Non-significant or significant at *p* ≤ 0.05, 0.01, and 0.001, respectively. Cultivar and harvest stage means were comparated by *t*-test. Interaction between the two factors were compared by Duncan’s multiple-range test (*p* = 0.05). Lower case letters indicate the significant differences of the interaction, upper case letters indicate the significant differences of the harvest stage mean effect.

**Table 3:** Linearity, LOD, LOQ, precision and recovery for the 16 authentic standards (*n* = 5).

Compound	LOD	LOQ	Linearity (R^2^)	Recovery % (*n* = 3)			Intra-Day Precision (RSD,%; (*n* = 3)	Intra-Day Precision (RSD,%; (*n* = 3)
	ng g^−1^			1 mg kg^−1^	10 mg kg^−1^	50 mg kg^−1^	1 mg kg^−1^	
Apigenin malonyl glucoside	0.04	0.12	0.991	91.3	99.5	105.3	5	6
Caffeic acid	0.05	0.14	0.992	99.3	91.6	102.3	3	8
Caffeic acid hexoside isomers	0.05	0.16	0.995	99.1	98.1	102.5	6	9
Caffeoyl feruloyl quinic acid	0.04	0.12	0.995	100.1	100.0	102.3	7	10
Chlorogenic acid	0.04	0.12	0.991	96.3	98.4	103.6	5	5
Coumaric acid	0.05	0.14	0.994	98.5	99.5	103.6	4	7
Coumaroyl quinic acid	0.04	0.13	0.994	99.1	100.6	102.2	2	8
Dicaffeoylquinic acid	0.04	0.12	0.995	100.2	99.5	104.6	7	9
Feruloyl quinic acid	0.03	0.10	0.991	100.6	99.9	104.5	5	7
Feruloyl tartaric acid	0.05	0.14	0.997	98.5	99.2	105.9	6	6
Quercetin malonyl glucoside	0.03	0.10	0.987	93.0	95.0	100.1	3	8
Quercetin-3-O-galactoside	0.05	0.14	0.992	99.9	100.1	102.5	5	9
Quercetin-3-O-glucoside	0.05	0.14	0.989	99.8	100.2	105.5	5	6
Quercetin-3-O-glucuronide	0.04	0.13	0.992	100.3	102.6	103.1	8	9
Rutin	0.04	0.12	0.991	102.9	99.0	101.8	6	9
Kaempferol-3-O-rutinoside	0.05	0.16	0.995	99.2	98.2	100.2	6	7

**Table 4:** Polyphenols content of green and red Salanova butterhead lettuce as influenced by harvest stage.

Polyphenols (µg g^−1^ fw)	Microgreens			Mature Leaves			Significance		
	Green	Red	*Mean*	Green	Red	*Mean*	Cultivar (C)	Harvest stage (H)	C × H
Apigenin malonil glucoside	17.14 ± 0.41 ^a^	5.19 ± 0.23 ^c^	*11.16 ± 2.68*	9.70 ± 0.4 ^b^	1.42 ± 0.12 ^d^	*5.56 ± 1.86*	***	ns	***
Caffeic acid	0.56 ± 0.01 ^c^	8.21 ± 0.05 ^a^	*4.39 ± 1.71*	0.32 ± 0 ^c^	3.14 ± 0.3 ^b^	*1.73 ± 0.64*	***	ns	***
Caffeic acid hexoside isomers	1.58 ± 0.03 ^a^	0.59 ± 0.01 ^c^	*1.09 ± 0.22*	0.97 ± 0.03 ^b^	0.39 ± 0 d	*0.68 ± 0.13*	***	ns	***
Caffeoyl feruloyl quinic acid	37.69 ± 0.02 ^b^	48.87 ± 0.37 ^a^	*43.28 ± 2.5 ^A^*	30.62 ± 0.02 ^d^	35.59 ± 0.17 ^c^	*33.11 ± 1.11 ^B^*	*	**	***
Chlorogenic acid	15.0 ± 0.34 ^c^	541 ± 29.5 ^a^	*278 ± 118*	6.92 ± 0.23 ^c^	234 ± 20.1 ^b^	*121 ± 51.7*	***	ns	***
Coumaric acid	0.93 ± 0.04 ^a^	0.49 ± 0 ^b^	*0.71 ± 0.1 ^A^*	0.51 ± 0 ^b^	0.34 ± 0.01 ^c^	*0.42 ± 0.04 ^B^*	*	*	***
Coumaroyl quinic acid	33.46 ± 0.55 ^b^	86.45 ± 0.65 ^a^	*59.96 ± 11.9 ^A^*	20.37 ± 0.51 ^c^	34.89 ± 0.34 ^b^	*27.63 ± 3.26 ^B^*	*	*	***
Dicaffeoylquinic acid	4.40 ± 0.04	18.14 ± 0.43	*11.27 ± 3.08*	nd	7.43 ± 0.35	*na*	*	na	na
Feruloyl quinic acid	1.97 ± 0.02 ^b^	3.15 ± 0.02 ^a^	*2.56 ± 0.26 ^A^*	0.84 ± 0.04 ^d^	1.35 ± 0.06 ^c^	*1.09 ± 0.12 ^B^*	ns	***	***
Feruloyl tartaric acid	nd	nd	*na*	58.62 ± 0.56	50.41 ± 0.04	*54.51 ± 1.85*	***	na	na
Kaempferol-3-O-rutinoside	4.91 ± 0.01	5.16 ± 0.09	*5.03 ± 0.07 ^A^*	3.23 ± 0.07	3.81 ± 0.06	*3.52 ± 0.14 ^B^*	ns	***	ns
Quercetin-3-O-galactoside	0.46 ± 0.01 ^c^	3.72 ± 0.02 ^a^	*2.09 ± 0.73*	0.33 ± 0 c	2.09 ± 0.11 ^b^	*1.21 ± 0.4*	***	ns	***
Quercetin-3-O-glucoside	0.62 ± 0.01 ^c^	4.22 ± 0.02 ^a^	*2.42 ± 0.8*	0.30 ± 0.01 ^d^	2.33 ± 0.02 ^b^	*1.31 ± 0.45*	***	ns	***
Quercetin-3-O-glucuronide	13.66 ± 0.29 ^a^	4.19 ± 0.1 ^c^	*8.92 ± 2.12*	5.43 ± 0.37 ^b^	2.64 ± 0.04 ^d^	*4.04 ± 0.65*	**	ns	***
Quercetin malonyl glucoside	94.4 ± 1.63 ^b^	228 ± 4.31 ^a^	*161 ± 29.9 ^A^*	45.1 ± 3.36 ^c^	94.8 ± 4.35 ^b^	*70.0 ± 11.38 ^B^*	*	*	***
Rutin	87.4 ± 0.59 ^b^	128 ± 1.36 ^a^	*108 ± 9.15 ^A^*	46.5 ± 1.84 ^d^	51.2 ± 1.19 ^c^	*48.9 ± 1.44 ^B^*	ns	***	***
Total polyphenols	314 ± 0.68 ^c^	1086 ± 25.8 ^a^	*700 ± 173*	230 ± 5.73 ^d^	526 ± 22.8 ^b^	*378 ± 67.1*	**	ns	***

All data are expressed as mean ± standard error, *n* = 3. ns,*,**, *** Non-significant or significant at *p* ≤ 0.05, 0.01, and 0.001, respectively. Cultivar and harvest stage means were comparated by *t*-test. Interaction between the two factors were compared by Duncan’s multiple-range test (*p* = 0.05). Lower case letters indicate the significant differences in the interaction, upper case letters indicate the significant differences of the harvest stage mean effect. nd: not detected, na: not applicable.

## Supplementary Materials

The following are available online at https://www.mdpi.com/2076-3921/9/4/300/s1, Table S1. Retention time and m/z ions selected for the identification and confirmation of the target compounds in the Orbitrap system; Figure S1. Green and red Salanova microgreens and matures leaves at harvest; Figure S2. Representative HPLC-Diode array chromatogram of carotenoids extracted from butterhead lettuce Salanova monitored at 450 nm; Figure S3. Representative Total Ion Chromatogram (TIC) of polyphenolic compounds extracted from butterhead lettuce Salanova evaluated by UHPLC-ESI-Orbitrap-MS.
